# Short and sweet: foreleg abnormalities in Havanese and the role of the FGF4 retrogene

**DOI:** 10.1186/s40575-020-00097-5

**Published:** 2020-12-07

**Authors:** Kim K. L. Bellamy, Frode Lingaas

**Affiliations:** 1 Department of Preclinical Sciences and Pathology, Faculty of Veterinary Medicine, Norwegian University of Life Sciences, P.O. Box 369 sentrum, N-0102 Oslo, Norway; 2 The Norwegian Kennel Club, P.O. Box 52 Holmlia, 1201 Oslo, Norway

**Keywords:** FGF4, Chondrodystrophy, Chondrodysplasia, Havanese, Short ulna, Genetic diversity, Exaggerations in conformation

## Abstract

**Background:**

Cases of foreleg deformities, characterized by varying degrees of shortened and bowed forelegs, have been reported in the Havanese breed. Because the health and welfare implications are severe in some of the affected dogs, further efforts should be made to investigate the genetic background of the trait.

A FGF4-retrogene on CFA18 is known to cause chondrodystrophy in dogs. In most breeds, either the wild type allele or the mutant allele is fixed. However, the large degree of genetic diversity reported in Havanese, could entail that both the wild type and the mutant allele segregate in this breed. We hypothesize that the shortened and bowed forelegs seen in some Havanese could be a consequence of FGF4RG-associated chondrodystrophy.

Here we study the population prevalence of the wild type and mutant allele, as well as effect on phenotype. We also investigate how the prevalence of the allele associated with chondrodystrophy have changed over time. We hypothesize that recent selection, may have led to a gradual decline in the population frequency of the lower-risk, wild type allele.

**Results:**

We studied the FGF4-retrogene on CFA18 in 355 Havanese and found variation in the presence/absence of the retrogene. The prevalence of the non-chondrodystrophic wild type is low, with allele frequencies of 0.025 and 0.975 for the wild type and mutant allele, respectively (linked marker).

We found that carriers of the beneficial wild type allele were significantly taller at the shoulder than mutant allele homozygotes, with average heights of 31.3 cm and 26.4 cm, respectively.

We further found that wild type carriers were born on average 4.7 years earlier than mutant allele homozygotes and that there has been a gradual decline in the population frequency of the wild type allele during the past two decades.

**Conclusions:**

Our results indicate that FGF4RG-associated chondrodystrophy may contribute to the shortened forelegs found in some Havanese and that both the wild type and mutant allele segregate in the breed. The population frequency of the wild type allele is low and appear to be decreasing. Efforts should be made to preserve the healthier wild type in the population, increase the prevalence of a more moderate phenotype and possibly reduce the risk of foreleg pathology.

## Plain English summary

Previous research and statements from owners, breeders and breed clubs, show that some Havanese have short and bowed forelegs. Most of these dogs show no signs of pain or discomfort, but a few of them do.

Some dog breeds are so-called chondrodystrophic, meaning that their legs are “too short” compared to the size of their body. Examples of chondrodystrophic breeds are dachshunds, bassets and corgis. The gene that causes chondrodystrophy is known and can be tested for (FGF4-retrogene on chromosome 18).

There is a lot of variation in the Havanese breed as regards color, size, head shape etc. We hypothesize that because there is so much variation in Havanese, it is possible that some of them are chondrodystrophic and some are not.

Could it be that Havanese with short and bowed forelegs do not have a “breed specific syndrome” as we have thought, but are simply chondrodystrophic?

We DNA-tested 355 Havanese to check this and to investigate whether things have changed over time. Is it possible that selection for certain conformational traits have unintentionally turned a primarily non-chondrodystrophic breed, chondrodystrophic, and subsequently made them more prone to foreleg bowing?

We found that some of the Havanese we DNA-tested are chondrodystrophic and some are not. In our sample, only about 5% of the dogs carry the non-chondrodystrophic gene variant.

We also found that carriers of the non-chondrodystrophic gene variant are taller at the shoulder than other Havanese, with average heights of 31.3 cm and 26.4 cm, respectively.

Carriers of the non-chondrodystrophic gene variant are born on average 4.7 years earlier than the other dogs in our sample. More Havanese are chondrodystrophic now, compared to two decades ago.

We recommend that Havanese are DNA-tested, to identify carriers of the non-chondrodystrophic gene variant. By breeding these dogs, we can prevent the variant being lost from the breed forever.

Carefully monitored outcrossings to non-chondrodystrophic individuals in closely related breeds may also be considered.

If we gradually increase the number of Havanese that are not chondrodystrophic, the breeds’ overall risk of foreleg problems will reduce, which would benefit the health and welfare of the breed.

## Background

Previous research has shown that foreleg deformities occur frequently in the Havanese breed [1]. In Norway, bowed forelegs is a common remark in dog show critiques and sporadic cases of short ulna syndrome have been reported [2]. In a survey conducted in the United States, 44% of Havanese owners replied that their dog had bowed, shortened or asymmetric forelegs [1].

Starr et al. [1] propose the idea of a breed specific syndrome in Havanese, including symptoms like bowed forelegs, cataracts, liver abnormalities and heart disease. Moderate heritability estimates were found and a few candidate genes were suggested [1].

Bowed forelegs in dogs is often a result of some form of leg shortening. When the growth of the long bones is stunted, it is often asynchronous as well. Disparity in length between the radius and ulna cause the shorter bone to act as a bowstring, which lead to the subsequent bowing of the longer bone. Stunted growth of the long bones, may be caused either by trauma to the growth plate before the dog is fully grown, or by genetic predisposition [3].

Several forms of hereditary disproportional dwarfism have been described in dogs [4–10]. A recessive mode of inheritance is reported in many breeds [4–10] and associated genes or possible causative mutations are known in some of them. A nonsense-mutation in the ITGA10-gene cause chondrodysplasia in Norwegian elkhounds and Karelian bear dogs [4]. In Labrador retrievers, a mild form of chondrodysplasia is associated with a mutation in the COL11A2-gene [6]. A deletion in the SLC13A1-gene has been associated with chondrodysplasia in miniature poodles [5].

In addition to breed specific forms of chondrodysplasia, disproportionally short legs also occurs as a desired and fixed trait in several dog breeds. Chondrodystrophy is caused by an expressed fibroblast growth factor 4 (FGF4) retrogene on chromosome 18, across dog breeds [11]. The FGF4-retrogene is responsible for the typical “short-legged” appearance of chondrodystrophic breeds like dachshunds, bassets and corgis.

Unlike chondrodysplasia, chondrodystrophy is often considered an accepted phenotypic variation, rather than a pathological condition. The trait is, however, still associated with increased risk of some health issues. Chondrodystrophic dogs are more likely to have bowed forelegs, and 3.5 times more likely to be affected with elbow disease, than non-chondrodystrophic dogs [12]. Angular limb deformity and elbow incongruity may cause abnormal strain on the joints and secondary degenerative joint disease [3]. In the chondrodystrophic dog breed Skye terrier, clear association was found between lameness and the degree of elbow incongruity [13].

Additionally, chondrodystrophic dog breeds are at increased risk of developing intervertebral disc disease [14], although recent research has shown that a FGF4-retrogene on CFA12 is of greater importance in intervertebral disc disease in dogs than the one on CFA18 [15, 16].

In the research that led to the discovery of the FGF4-retrogene on CFA18, four breeds (jack russel terrier, west highland white terrier, Havanese and Sussex spaniel) were excluded from the initial association analyses because leg length in these breeds was uncertain or variable. Later, sequencing of the insert revealed that out of seven Havanese included in the original study, six were homozygote for chondrodystrophy and one was heterozygote. The authors air the idea that the previously reported “Havanese syndrome” may disguise the absence of the retrogene and that this could be the reason that the trait is not fixed [11].

The Havanese breed was created from various small dogs and anecdotally there was significant conformational variation in the founder dogs that is still evident [17]. Several reports also show a relatively high degree of heterozygosity in the breed [18, 19]. It is plausible, that contrary to the situation in most other breeds, both alleles of the FGF4-retrogene segregate in this breed. We hypothesize that the short and bowed forelegs seen in some Havanese could potentially be a result of chondrodystrophy, rather than a breed specific syndrome as previously suggested.

The prevalence of bowed and shortened forelegs in the Havanese breed is high [1, 2]. Although most cases show little signs of discomfort or pain, the negative effect on health and welfare is severe in some cases. If the shortened and bowed forelegs seen in Havanese are directly associated with chondrodystrophy, increasing the population frequency of the non-chondrodystrophic allele, could reduce the breeds overall risk of foreleg pathology.

The aim of this study was to investigate the presence/absence of the chondrodystrophic genotype in the Norwegian population of Havanese dogs, as well as its effect on phenotype. We also studied how the population frequency of the wild type and mutant allele has changed over time.

## Results

### Prevalence

We genotyped an A/G SNP on chromosome 18 (CFA18), base position 23,432,408 (CanFam2), that has previously been reported as part of a “chondrodystrophy-haplotype” [11], for a random sample of 355 Havanese. We found that although most individuals were homozygote for the allele associated with chondrodystrophy (A), 5% of the population carried one copy of the wild type allele (G). The allele frequencies were 0.975 and 0.025 for the chondrodystrophy-associated allele and the wild type allele, respectively. No dogs were homozygote wild type.

To verify the linkage disequilibrium between the marker and the insert, 22 A/A-dogs and 22 A/G-dogs were assayed for the FGF4 insertion on CFA18. The LD between the SNP and the causative insert was complete in our sample (*n* = 44).

### Association

**Fig. 1:**
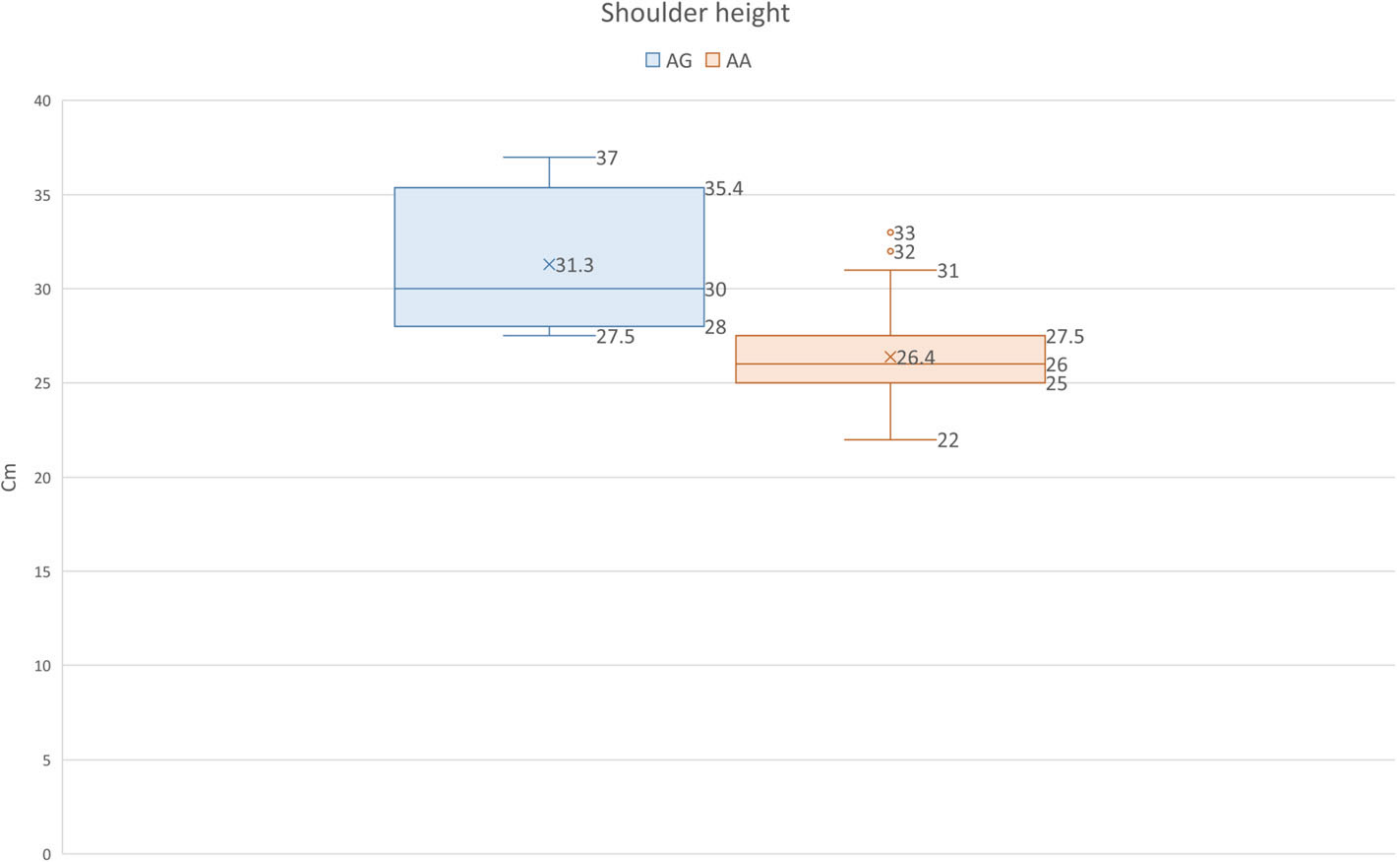
Average and median shoulder height in genotype groups AG and AA

We found significant association between genotype and shoulder height in Havanese (*n* = 103). Havanese with one copy of the beneficial allele (A/G) were on average 4.9 cm taller than risk allele homozygotes (A/A) (*p* < 0.0001), with an average heights of 31.3 cm and 26.4 cm, respectively (Fig. 1).

### Change in allele frequency over time

**Fig. 2:**
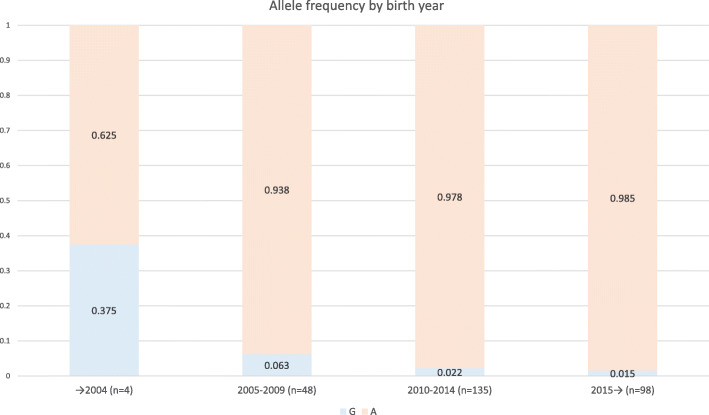
Allele frequencies for the wild type (G) - and mutant (A) allele, by birth year

To investigate potential changes over time, we genotyped a random sample of 285 Havanese with available information on birth year. Havanese that carried the wild type allele were born on average 4.7 years earlier than A/A-homozygotes (*p*-value < 0.0001). Analysis of allele frequencies in different birth year groups, show that there has been a gradual decline in the allele frequency of the wild type allele during the past two decades (Fig. 2).

## Discussion

We show that both the wild type and mutant allele of the FGF4-retrogene segregate in the Norwegian population of Havanese dogs and that it is associated with shoulder height. Our results support that the short and bowed forelegs seen in some Havanese could potentially be a result of chondrodystrophy, rather than a breed specific syndrome as previously suggested.

It should be noted, that the prevalence of the risk allele is high, but the number of severely affected individuals (e.g. those requiring surgery) is low, which means that modifying genes probably affect the degree of foreleg bowing and elbow incongruity in the chondrodystrophic dogs. Our result does not uncover other associated genes, but highlight the increasing population frequency of an unnecessary, underlying risk factor.

For most dogs in the study, we genotyped a very closely linked variant rather than the causative insert itself. The studied SNP is, however, located only 1272 base pairs away from the insert site (~ 0.001 cM), which means the likelihood of a recombination is very low. We have verified a complete LD between the variant and the retrogene in a selection of 44 Havanese with genotypes A/G (*n* = 22) and A/A (*n* = 22). The strong association between the marker and phenotype also point towards true variation in the presence/absence of the retrogene.

Shoulder height was selected as a phenotypic marker for foreleg shortening, because it could be easily and reliably measured by the owner. A more standardized measure, e.g. using radiographs to evaluate the degree of foreleg bowing or having one person measure all the dogs, could have improved precision of the measurements, but would significantly reduce the number of dogs we were able to include in the study. We believe the degree of error in owner measurements is similar in the two genotype-groups and should therefore not affect the result of our association analysis.

The primary aim of the study was to investigate the frequency of the FGF4 retrogene and discuss potential effects on the population risk of foreleg pathology. A thorough clinical evaluation of the dogs, which would be necessary to accurately classify the degree of foreleg bowing and give a conclusive description of prevalence, was beyond the scope of this study. All owner-reported cases of severe foreleg bowing have been from dogs that are risk allele homozygotes.

We did not identify any wild type homozygotes. This is not surprising, given the low population frequency of the wild type allele. The absence of G/G-individuals prevent us from investigating possible phenotypic differences between G/G-dogs and heterozygotes. Traditionally, chondrodystrophy has been considered a dominant trait in dog, but the significant height difference we found between A/G- and A/A-individuals show that at least in this breed, the dominance is incomplete. Some forms of chondrodysplasia in human, also show incomplete dominance [20].

The Fédération Cynologique Internationale (FCI) breed standard for Havanese [21], states that the height at the withers should be between 23 cm and 27 cm (tolerance 21 cm to 29 cm), which means the average height of the A/A-dogs is correct. Increasing the number of A/A- x A/G-matings, would reduce the prevalence of dogs with disproportionally short legs, with a risk that some offspring might be too tall according to standard. We believe that preserving the wild type allele before it is lost should be of high priority. We therefore suggest allowing a limited increase in height for the first generations that may be corrected in succeeding generations through traditional selection.

A slight increase in the height acceptance in the breed standard could also be considered. This would allow a faster change in allele frequency and still leave room to focus on other traits, because the need to select for height would decrease. Increasing the height acceptance to 30 cm, which equals the median height of the A/G-dogs, would be enough to ensure most A/G-dogs are still within standard. This is also in accordance with what some consider to be the original, Cuban standard [17].

Lastly, it should be noted that the standard lists a “French front” (pasterns to close and feet turned outwards) as an important fault [21].

We show a decline in the population frequency of the wild type allele during the past two decades, with A/G-dogs being on average 4.7 older than A/A-dogs. This finding is supported by statements from breeders, who indicate that there has been a “trend” of selection for longer backs and shorter legs in recent years. It is possible that a selection for certain conformational traits have unintentionally turned a primarily non-chondrodystrophic breed, chondrodystrophic.

Chondrodystrophy is associated with increased risk of angular limb deformity and elbow disease [12]. If the shortened and bowed forelegs seen in Havanese are directly associated with chondrodystrophy, increasing the prevalence of the non-chondrodystrophic wild type in the population could reduce the number of dogs with increased risk of foreleg pathology, subsequently reducing the number of clinically affected individuals. This would benefit the health and welfare of the breed.

Marker-assisted selection should be implemented to gradually increase the population frequency of the beneficial allele and ensure that the non-chondrodystrophic type is not lost. We believe any increase in the frequency of the wild type allele has the potential to reduce risk of foreleg pathology and that ideally, the wild type should eventually become be the predominant variant. However, it is challenging to obtain a fast change in allele frequency without negatively influencing genetic variation and/or other traits. The initial goal should therefore be to recover a sustainable population of non-chondrodystrophic individuals and avoid that the risk allele becomes fixed.

DNA-testing as many Havanese as possible for the FGF4-retrogene on CFA18, would be valuable to identify the rare, wild type carriers for breeding purposes. Litters from wild type carriers should be tested prior to adoption, to ensure continuation of the breeding program.

To avoid loss of genetic variation through selection for the low frequency wild type, it may also be worth considering a limited outcross to wild type carriers in closely related breeds like the bichon frisé. If done right, such an outcross could increase the prevalence of the wild type allele and speed up the reversal process, without much negative effect on other traits because the breeds are so similar.

Parallel to breeding for a gradual increase in the population frequency of the non-chondrodystrophic genotype, efforts should be made to reduce the degree of foreleg deformities and elbow incongruity among the chondrodystrophic Havanese. Selection response in other chondrodystrophic breeds have shown that it is possible to reduce the degree of foreleg bowing by selection based simply on visual inspection. A suggested protocol for classification of elbow incongruity in chondrodystrophic breeds [13], could potentially be used to screen chondrodystrophic Havanese prior to breeding.

## Conclusions

Our findings show that leg length in Havanese is strongly associated with FGF4-retrogene variants, in an incomplete dominant manner. The allele frequency of the wild type allele is low and appear to be decreasing. Efforts should be made to preserve the healthier wild type allele in the population, increase the prevalence of a more moderate phenotype and reduce the risk of foreleg pathology.

## Methods

### Dogs

Two batches of samples, all collected with owners’ consent, were included in the study. The first batch of samples was recruited specifically for this project, for the association analysis. Owners were asked to measure the shoulder height of their dog and send in a cheek swab for DNA-studies (*n* = 120). The samples were collected using Performagene™ buccal swabs (DNA Genotek Inc), administered by the owner. DNA was extracted following the manufacturer’s recommendations. The second batch of samples was originally recruited for a research project on behaviour [22] and was readily available through our DNA biobank (*n* = 235). The second batch of dogs was only included in the allele frequency calculation and birth year analyses. The only inclusion criteria in both batches were age > 1 year old and that the owner was willing to participate. DNA was stored at − 20 degrees Celsius.

### Genotyping

An A/G SNP at base position CFA18:23432408 (CanFam2), that has previously been reported as part of a “chondrodystrophy-haplotype”, was genotyped for 355 Havanese. The SNP is positioned 1272 base pairs downstream of the insert [11]. Primers used were forward: ‘TTACCCACAAGGAAGATACAGC’ [11] and reverse: ‘TGCAGTGACCCCATCAGTTC’. Primer3plus was used to create the reverse primer. Sequencing of the PCR products were performed following a standard Sanger method on an ABI 3500 XL DNA analyzer (Applied Biosystems, Life Technologies of Thermo Fisher Scientific), followed by manual inspection using the Sequencher software from Gene Codes Corporations.

Linkage disequilibrium between the SNP and the causative insert was checked and verified in a material of 44 dogs. We amplified the insert site on CFA18 in Havanese with genotypes A/A (*n* = 22) and A/G (n = 22) (G/G not available), using allele-specific PCR. Primers used were: forward: F_flank: ‘TTGGGAATGTCAAACCACTG’, F_insert ‘GTCCGTGCGGTGAAATAAAA’ and reverse: R_flank: ‘GTTCCCTCCATTTCGGTTT’ [23]. When no insert was present, the primers F_flank/R_flank gave a PCR-product~ 388 bp. When an insert was present, the primers F_insert/R_flank gave a PCR-product~ 168 bp. Following the PCR reaction, results were visualized by gel electrophoresis and manual inspection.

The allele frequencies were calculated using the formula: *p* = *f* (AA) + 0.5 *f* (AG), q = *f* (GG) + 0.5 *f* (AG).

### Association analyses and statistics

Shoulder height measured by the owner, was selected as a phenotypic marker for the degree of foreleg shortening. Shoulder height was defined as the distance from the ground to the “withers”, i.e. the ridge between the shoulder blades at the tallest part of the dogs back, near the base of the neck.

For the association analyses on shoulder height and birth year, the mean and standard deviation for each genotype was calculated in Excel (AVERAGE, STDEV.S). The pooled standard deviation and standard error, as well as the significance level using the t-test, were calculated using MedCalc [24].

## Data Availability

The dataset analyzed during the current study is not publicly available due to difficulty in fully anonymizing the individual dogs, but is available from the corresponding author on reasonable request.
